# Progression analysis versus traditional methods to quantify slowing of disease progression in Alzheimer’s disease

**DOI:** 10.1186/s13195-024-01413-y

**Published:** 2024-02-29

**Authors:** Linus Jönsson, Milana Ivkovic, Alireza Atri, Ron Handels, Anders Gustavsson, Julie Hviid Hahn-Pedersen, Teresa León, Mathias Lilja, Jens Gundgaard, Lars Lau Raket

**Affiliations:** 1 Division of Neurogeriatrics, Department of Neurobiology, Care Sciences and Society, Karolinska Institutet, Solna, 171 64 Sweden; 2 Novo Nordisk A/S, Søborg, Denmark; 3 Faculty of Health, Medicine and Life Sciences, School for Mental Health and Neuroscience, Department of Psychiatry and Neuropsychology, Alzheimer Centre Limburg, Maastricht University Medical Centre+, Maastricht, MD 6200 The Netherlands; 4 Quantify Research, Hantverkargatan 8, Stockholm, 112 21 Sweden; 5 Clinical Memory Research Unit, Department of Clinical Sciences, Lund University, Lund, Sweden; 6 Banner Sun Health Research Institute and Banner Alzheimer’s Institute, Banner Health, Sun City and Phoenix, AZ USA; 7 Center for Brain/Mind Medicine, Department of Neurology, Brigham and Women’s Hospital, Harvard Medical School, Boston, MA USA

**Keywords:** Alzheimer’s disease, Disease progression, Statistical model

## Abstract

**Background:**

The clinical meaningfulness of the effects of recently approved disease-modifying treatments (DMT) in Alzheimer’s disease is under debate. Available evidence is limited to short-term effects on clinical rating scales which may be difficult to interpret and have limited intrinsic meaning to patients. The main value of DMTs accrues over the long term as they are expected to cause a delay or slowing of disease progression. While awaiting such evidence, the translation of short-term effects to time delays or slowing of progression could offer a powerful and readily interpretable representation of clinical outcomes.

**Methods:**

We simulated disease progression trajectories representing two arms, active and placebo, of a hypothetical clinical trial of a DMT. The placebo arm was simulated based on estimated mean trajectories of clinical dementia rating scale–sum of boxes (CDR-SB) recordings from amyloid-positive subjects with mild cognitive impairment (MCI) from Alzheimer’s Disease Neuroimaging Initiative (ADNI). The active arm was simulated to show an average slowing of disease progression versus placebo of 20% at each visit. The treatment effects in the simulated trials were estimated with a progression model for repeated measures (PMRM) and a mixed model for repeated measures (MMRM) for comparison. For PMRM, the treatment effect is expressed in units of time (e.g., days) and for MMRM in units of the outcome (e.g., CDR-SB points). PMRM results were implemented in a health economics Markov model extrapolating disease progression and death over 15 years.

**Results:**

The PMRM model estimated a 19% delay in disease progression at 18 months and 20% (~ 7 months delay) at 36 months, while the MMRM model estimated a 25% reduction in CDR-SB (~ 0.5 points) at 36 months. The PMRM model had slightly greater power compared to MMRM. The health economic model based on the estimated time delay suggested an increase in life expectancy (10 months) without extending time in severe stages of disease.

**Conclusion:**

PMRM methods can be used to estimate treatment effects in terms of slowing of progression which translates to time metrics that can be readily interpreted and appreciated as meaningful outcomes for patients, care partners, and health care practitioners.

**Supplementary Information:**

The online version contains supplementary material available at 10.1186/s13195-024-01413-y.

## Background

Alzheimer’s disease (AD) is a debilitating neurodegenerative disorder and a primary cause of dementia resulting in reduced life expectancy [1, 2], loss of function and autonomy [1, 3], impaired quality of life (QoL) [4], care partner burden [5–7], and high costs to society [8, 9]. Recent estimates suggest that 32 million people have dementia due to AD worldwide and 69 million mild cognitive impairment (MCI) due to AD [10].

Up until recently, the management of AD has been limited to symptomatic treatment and supportive care [11–13], in addition to non-pharmacological multidomain lifestyle-based prevention strategies [14]. Recently, two disease-modifying treatments (DMTs), aducanumab and lecanemab, received accelerated approval in the United States (US) by the Food and Drug Administration (FDA) [15, 16]; however, the Centers for Medicare & Medicaid Services (CMS) restricted its coverage to patients enrolled in approved clinical trials [17, 18]. More recently, in July 2023, lecanemab received traditional FDA approval, and subsequently, CMS made a national coverage determination to provide treatment coverage for patients enrolled in a CMS-approved registry for real-world data collection [19]. A third DMT, donanemab, is currently under FDA review for traditional approval. These important developments are expected to have large impacts on the diagnostic and management paradigms for AD which will require major system-wide changes and induce logistical challenges [20].

Many AD experts view the observed group-level efficacy demonstrated in clinical trials for these first-in-class DMTs as both foundational therapeutic steps to build on and of potential to provide meaningful benefits for treated individuals [21–24]. However, some also question the clinical value or meaningfulness of observed treatment effects when also considering potential safety risks and the costs and inconvenience associated with frequent treatment administration and safety monitoring [25–27]. The concept of clinical meaningfulness is central to this discussion. While perhaps being a broad and not easily defined concept [23], for the purpose of this paper, we consider clinical meaningfulness to describe the perceived meaning or value of observed effects from the perspective of primarily patients but also their families and other key stakeholders.

The assessment of clinical meaningfulness of DMTs in AD is challenging for two main reasons. First, the benefits of DMTs that impact one aspect of AD (e.g., amyloid removal) are expected to build over time and become most pronounced over the long term. The slowly progressing nature of clinical symptoms in early-stage AD makes typical trial durations of 18–24 months of follow-up too short to show larger effect sizes that accrue on long-term clinical outcomes. Benefits from DMTs would be expected to increasingly accumulate and manifest better over longer intervals than 2 years in individuals with early-stage clinical AD [23]. Second, meaningful benefits represent a latent trait, the manifestations of which depend on several perspectives (e.g., patient-centric, caregiver-centric, clinician-centric, clinical trialist-centric, regulatory agency-centric, health system-centric, payer-centric) and different measures that assess multiple domains, states, or goals. The absence of purely objective and accepted observable events (such as myocardial infarction, stroke, or fracture in other chronic diseases), or clinically validated surrogate biomarkers, makes it more challenging to interpret the meaningfulness of trial results using only one measure or perspective [28, 29]. Instead, cognitive and functional assessment batteries are used to elicit the severity of symptoms as proxies for the staging of disease, and also as clinical outcomes in clinical trials. However, these typical clinical trial outcome measures can be heterogeneous, lack adequate sensitivity to measure change in disease progression, and may not reflect what patients and other key stakeholders value the most [30], ultimately resulting in greater uncertainty regarding the value and meaningfulness of interventions. Assessments of QoL are often included as secondary endpoints in clinical trials [31]. While they may be helpful in assessing the meaningfulness of observed short-term effects, they do not necessarily capture the full value of treatment, and especially not over the long term.

The clinical dementia rating (CDR) scale, and specifically its sum of boxes score (CDR-SB), is one of the most commonly used clinical trial measures for assessing treatment efficacy on cognitive-functional severity in early-stage clinical AD (i.e., MCI and mild dementia in AD). Efforts to delineate effect sizes that can be considered clinically meaningful when analyzing change from baseline in CDR-SB have remained uncertain and contested due several factors, including differences in definitions, methodology, populations and study-specific characteristics such as clinical trials versus observational studies, inclusion/exclusion criteria (including stage and biomarker validation), assessment intervals, and protocol and rater-related characteristics, all of which impact signal-to-noise ratios and detection and appreciation of sensitivity to changes [32–35].

Symptomatic treatments in AD are standard-of-care and provide value, however, they are not expected to change the long-term slope of clinical decline, and if, or when, discontinued would not be expected to produce persistent benefits [13]. DMTs, however, are postulated to produce treatment effects that persist and even accumulate over time resulting in a long-term delay of disease progression, which if sizeable surely would be considered clinically meaningful. At least part of their effect is expected to persist also after the discontinuation of treatment. However, the value of lower scores on clinical outcome measures (e.g., CDR-SB) at different stages of dementia severity and over time, particularly on intervals of 1–2 years, also remain opaque and not well-understood, especially by clinicians and patients and families, as these outcomes (e.g., CDR scale) are not used in clinical practice. Therefore, when expressing the effect of potential DMTs on clinical outcomes as a time delay or relative slowing of progression could offer a powerful, face-valid, and readily interpretable (by researchers, clinicians, and patients and families alike) alternative to reporting a typical change in points on a scale such as CDR-SB. This should not be seen as a substitute to standard methods, but rather as a completement with specific merits.

In this work, we demonstrated how a new statistical method, progression models for repeated measures (PMRM), can be used to estimate a slowing of disease progression from simulated trial data of a hypothetical DMT in AD [36]. We also show how estimates of slowing of progression from clinical trials can be implemented in a standard Markov model, commonly used for the health economic evaluation of treatments in AD [37, 38]. We explore whether estimating in the time dimension the slowing of progression can both add to the interpretability of a potential clinical treatment effect and offer additional statistical power by making the best use of the available data.

## Methods

### Concept of PMRM

PMRM are a new class of flexible nonlinear mixed-effects models that enables estimation of the treatment effect in terms of the slowing or delay of the time in disease progression. They are particularly applicable in progressive diseases such as AD and Parkinson’s disease, where potential DMTs may delay or slow disease progression on a commonly used outcome scale relative to the placebo group. In addition to enabling estimation of time delays and the associated relative slowing of disease progression, the PMRM framework also provides low-dimensional parameterizations of treatment effects to estimate the absolute or proportional reduction in clinical decline [23, 36].

The PMRM is a novel method, but time-based differences in general have already been possible to estimate using alternative model specifications, such as with accelerated failure time (AFT) models. The AFT is a parametric model where estimates of time-based differences are possible since the estimated parameters of the AFT model measure the effect of a particular covariate on the mean/median survival time. AFT models use time-to-event data and have been widely used in some fields, e.g., within oncology and aging [39, 40]. The PMRM can be seen as an extension of the AFT framework to model a continuous outcome measure (rather than a single event).

### Case study

#### Simulation

To simulate a realistic interventional trial, testing the effects of a potential DMT in AD, data was obtained from the Alzheimer’s Disease Neuroimaging Initiative (ADNI) [41]. ADNI was launched in 2003 and has recruited/collected data/specimens from more than 1700 participants with unimpaired cognition, significant memory concern, MCI, and dementia due to AD in the US and Canada. The main objective of ADNI has been to assess if the clinical and neuropsychological data, neuroimaging data, genetic data, and data related to biochemical biomarkers can be combined to determine the progression of MCI and dementia due to AD [41, 42]. Up-to-date information on ADNI is available at https://adni.loni.usc.edu/.

#### Inclusion criteria

The placebo arm of a clinical trial in amyloid-positive MCI subjects was simulated using the estimated disease trajectories from ADNI participants meeting typical clinical trial inclusion criteria. ADNI participants were selected if they were amyloid positive according to a brain positron emission tomography scan or analysis of cerebrospinal fluid, aged ≥ 55 to < 86 years, and had a clinical diagnosis of MCI, which was defined as having a score ≥ 22 on the Mini-Mental State Examination (MMSE, range 30–0, higher scores indicate less impairment) [43] and a CDR-SB score < 4.5, at baseline. A total of 537 participants met these inclusion criteria. Baseline characteristics for the included ADNI participants are shown in Table 1.

**Table 1:** Baseline characteristics for 537 participants included from ADNI^a^

*N*	Mean age, years	Male, *n*	Median CDR-SB	Median MMSE
537	72.7	313	1.5	28

^a^This cohort was used to simulate a placebo arm of a clinical trial in amyloid-positive MCI patients. The CDR scale is a staging tool used to determine the severity of dementia-related symptoms across six domains (three cognition domains: memory, orientation, judgment & problem solving; three functional domains: community affairs, home & hobbies, and personal care) each of which is scored between 0 and 3 following a semi-structured interview. The CDR-SB score is the sum of all six domain scores (range 0–18) and is considered a more sensitive measure of dementia severity compared to the alternative CDR-Global score [44]

#### Primary outcomes

Primary outcomes were defined to be CDR-SB for the comparison of models where the outcome is measured on a continuous scale, and time to progression to dementia derived by dichotomizing CDR-SB for the time-to-event model. Progression to dementia was defined as reaching a CDR-SB ≥ 4.5 at a post-baseline visit, corresponding to transitioning into mild dementia as suggested by O’Bryant et al. (2010) [45].

**Fig. 1:**
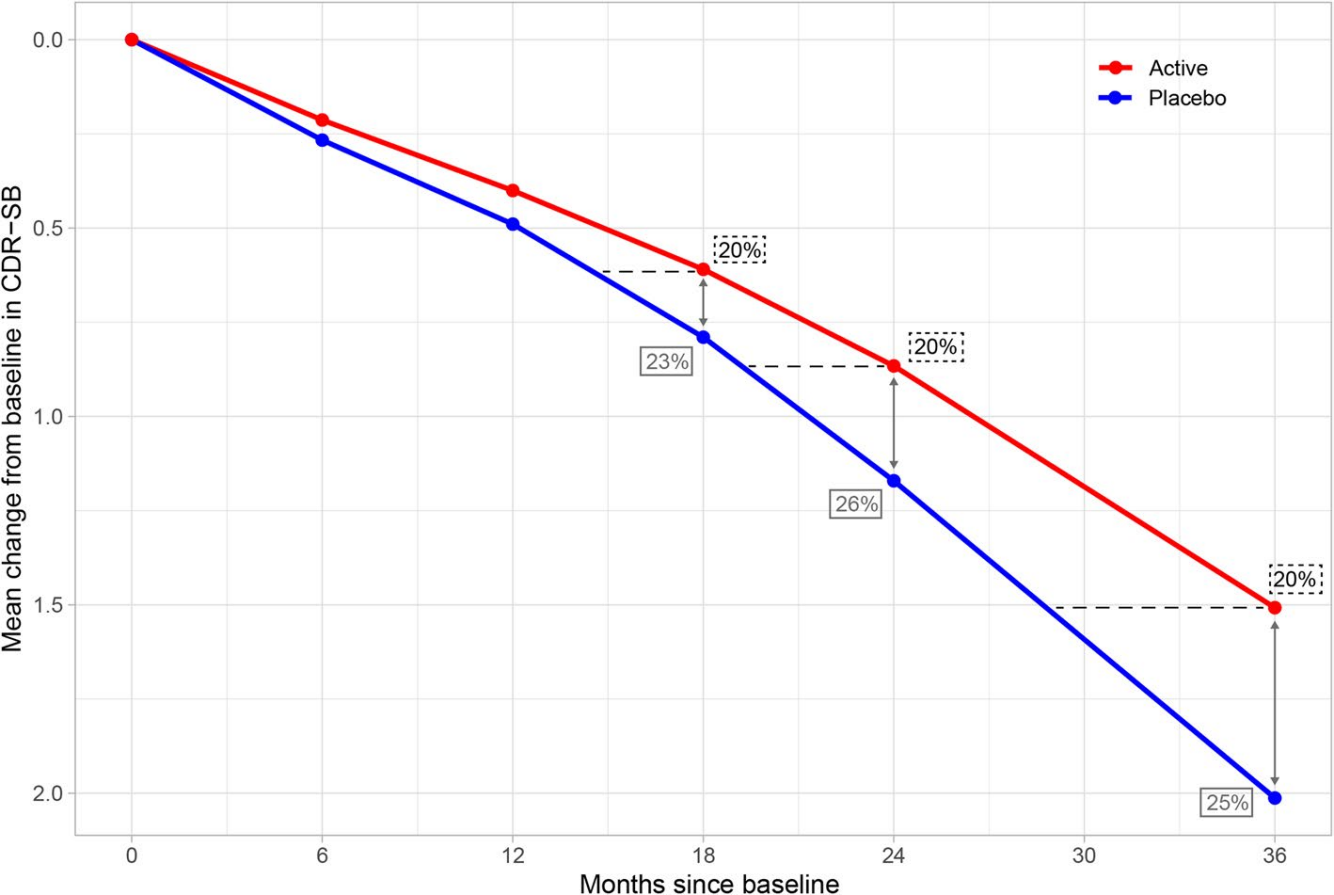
Estimated mean trajectory of the ADNI participants’ data (placebo) and the corresponding active treatment arm. The active treatment arm is derived by applying a proportional 20% time delay in disease progression at each visit compared to placebo (dashed horizontal lines)

The CDR-SB trajectory was estimated using a constrained longitudinal data analysis model with assumed identical mean CDR-SB across treatment arms due to randomization and an unconstructed covariance matrix (meaning no assumptions are made about the variances and covariances between an individual’s scores across visits) [46]. Data from baseline visits, and at months 6, 12, 18, 24, and 36 after baseline were used, and the estimated mean values across the six visits, corresponding to the placebo arm, are shown in Fig. 1. Tabular data and the estimated covariance matrix describing the variation across visits are presented in the Additional file. A 20% slowing of disease progression was applied to the mean placebo trajectory at each visit using linear interpolation to simulate the mean trajectory of the active treatment arm (Fig. 1). A time delay may be better aligned with a disease-modifying effect thought to delay disease progression. It may also be easier to interpret an expected delay of the disease by *X* months compared to a reduction of *Y* points on a clinical scale. Finally, a reduction in progression implicitly assumes treated patients will never progress to the same point as comparators, whereas the time delay will still allow all to fully decline in due time.

Participant-level trajectories were simulated based on estimated CDR mean trajectories and a temporal correlation structure. For the time-to-event model, the simulated participant-level trajectories were later dichotomized into the progression to dementia outcome. A thousand simulations were conducted across a number of trial scenarios, varying the number of patients per arm (300, 400, 500, 600, and 700 individuals) and trial duration (18, 24, and 36 months). Further details of the simulation are described in the Additional file.

### Statistical models

The present study included time-based PMRM [36], mixed models of repeated measures (MMRM) [46] as well as the Cox proportional hazard model [47].

The time-based PMRM is a flexible model from the PMRM family of models for estimating time-based changes in disease progression without assuming proportional slowing across visits. The time-based PMRM assumes the mean outcome of active treatment can be described as the mean outcome of the placebo group at a different time. The DMT treatment effect estimated at each visit represents the slowing of disease progression in the active arm relative to the placebo arm (as shown by the dashed lines in Fig. 1). For example, the treatment effect at the final visit in Fig. 1 corresponds to a 20% slowing of disease progression in the active arm compared to the placebo arm, which translates to a 7-month slowing of progression.

The MMRM are a class of statistical models often used in studies with longitudinal continuous outcomes to account for the correlation between repeated measurements within each patient [48]. In contrast to the PMRM, which measures the treatment effect on the unit of time, the MMRM measures the treatment effect on the unit of the outcome (corresponding to the *y*-axis in Fig. 1).

Cox proportional hazards model is a widely used semi-parametric model to estimate the relationship between covariates and the time to an event. The estimates of the Cox model are often presented in terms of hazard ratios which can be interpreted as the risk of an event relative to exposure, e.g., as the reduction in the risk of progressing from MCI to dementia due to treatment [49].

### Implementation in a health economic model

To estimate the value of a treatment, one needs to map the estimate of treatment effect from short-term clinical trials to a set of measures that can be ascribed long-term value through a health economic model. Here we translate the slowing of disease progression, as estimated by PMRM from trial data, to estimates of long-term impact on clinical trajectories using a Markov model. This type of model has commonly been used for the health economic evaluation of treatments in AD [37, 38]. Markov models divide patients into a finite number of states and examine how patients transition between these states over time. Each state is typically assigned relevant outcomes such as costs and QoL. Treatments are modeled by changing the probabilities of making transitions between states. The purpose of the modeling is to examine how treatment changes the amount of time spent in each of the model states, and how this translates into changes in outcomes such as costs and QoL. Our analysis is limited to looking at time in each state. The Markov model was run in monthly cycles with a horizon of 15 years, simulating transitions between four states: MCI due to AD, mild AD dementia, moderate-to-severe AD dementia, and death. Constant transition intensities were applied over all cycles of the model. Mortality was approximated at a monthly probability of death at 0.3%, 0.5%, and 1.0% in MCI due to AD, mild AD dementia, and moderate-severe AD dementia respectively, which corresponds to the expected risk of death in a US 70-year-old assuming a relative risk increase of 2, 3, and 6 in each state, respectively [50]. Transitions between alive states were adapted using previously published data from beta-amyloid-positive individuals in the National Alzheimer’s Coordinating Center database [50, 51] assuming no back-transitions to less severe states. That is, annual probabilities of 23% from MCI due to AD to mild AD dementia and 39% from mild AD dementia to moderate-to-severe AD dementia, (the residuals remaining in the same state) [50]. Annual probabilities were transformed to monthly probabilities by the traditional approach [52], and half-cycle correction was applied to the first and last cycle.

We simulated two arms in the model: one on active treatment and one on placebo. The standard approach of simulating a treatment effect in a Markov model is to apply a risk reduction, e.g., as estimated by a Cox model, on the transition probabilities to more severe stages of disease. This corresponds to an effect on the *y*-axis showing the proportion in each state at a specific point in time (e.g., in Fig. 1). Instead, when implementing a slowing of disease progression, we extended the time axis (i.e., the *x*-axis) of the treatment arm by the estimated percentage slowing. Thereby the simulated treatment arm has the same distributions across states as the placebo arm at each cycle, but the cycle lengths of the treatment arm are longer.

## Results

### Estimated treatment effects

The change from baseline in CDR-SB was estimated using the treatment effects from the fitted PMRM and the MMRM with 700 patients across three scenarios with trial lengths of 18, 24, and 36 months, respectively. To evaluate the power of the different models for estimating treatment effects, 1000 simulations of each scenario were replicated. The median treatment effects are shown in Table 2. The PMRM estimated a 19% horizontal delay in disease progression in the scenario with a trial length of 18 months, 19% with 24 months, and 20% with 36 months. This translates to a delay of progression of ~ 7 months over a trial duration of 36 months. The MMRM estimated a 22% vertical reduction in CDR-SB in the scenario with an 18-month trial length and a 25% reduction in scenarios with trial lengths of 24 and 36 months, corresponding to approximately a 0.5-point worsening in CDR-SB in the placebo arm compared to active treatment after 36 months. The risk of progressing to dementia during the study period was estimated using the Cox proportional hazards model, which found a hazard ratio of 0.85 of the risk of progressing to dementia in the scenario with an 18-month trial length, and a hazard ratio of 0.83 in scenarios with trial lengths of 24 and 36 months.

**Table 2:** Median treatment effects estimated by the PMRM, MMRM, and the Cox proportional hazards model^a^

Sample size	Study length (months)	Median effect		
		**PMRM (% time delay, measured on the horizontal axis)**	**MMRM (% reduction in CDR-SB, measured on the vertical axis)**	**Cox (HR of progressing to dementia)**
200	18	19%	23%	0.84
200	24	20%	26%	0.82
200	36	20%	25%	0.82
300	18	19%	23%	0.85
300	24	19%	26%	0.83
300	36	20%	25%	0.84
400	18	19%	22%	0.85
400	24	20%	26%	0.83
400	36	20%	25%	0.83
500	18	19%	22%	0.85
500	24	20%	26%	0.83
500	36	20%	25%	0.83
600	18	19%	22%	0.85
600	24	19%	26%	0.83
600	36	20%	25%	0.83
700	18	19%	22%	0.85
700	24	19%	25%	0.83
700	36	20%	25%	0.83

^a^Data are shown across scenarios with different sample sizes and study lengths (1000 simulations per scenario). The table shows the treatment effect at the final visit for the PMRM and the MMRM. The hazard ratio summarizes the treatment effect over the entire study duration

**Fig. 2:**
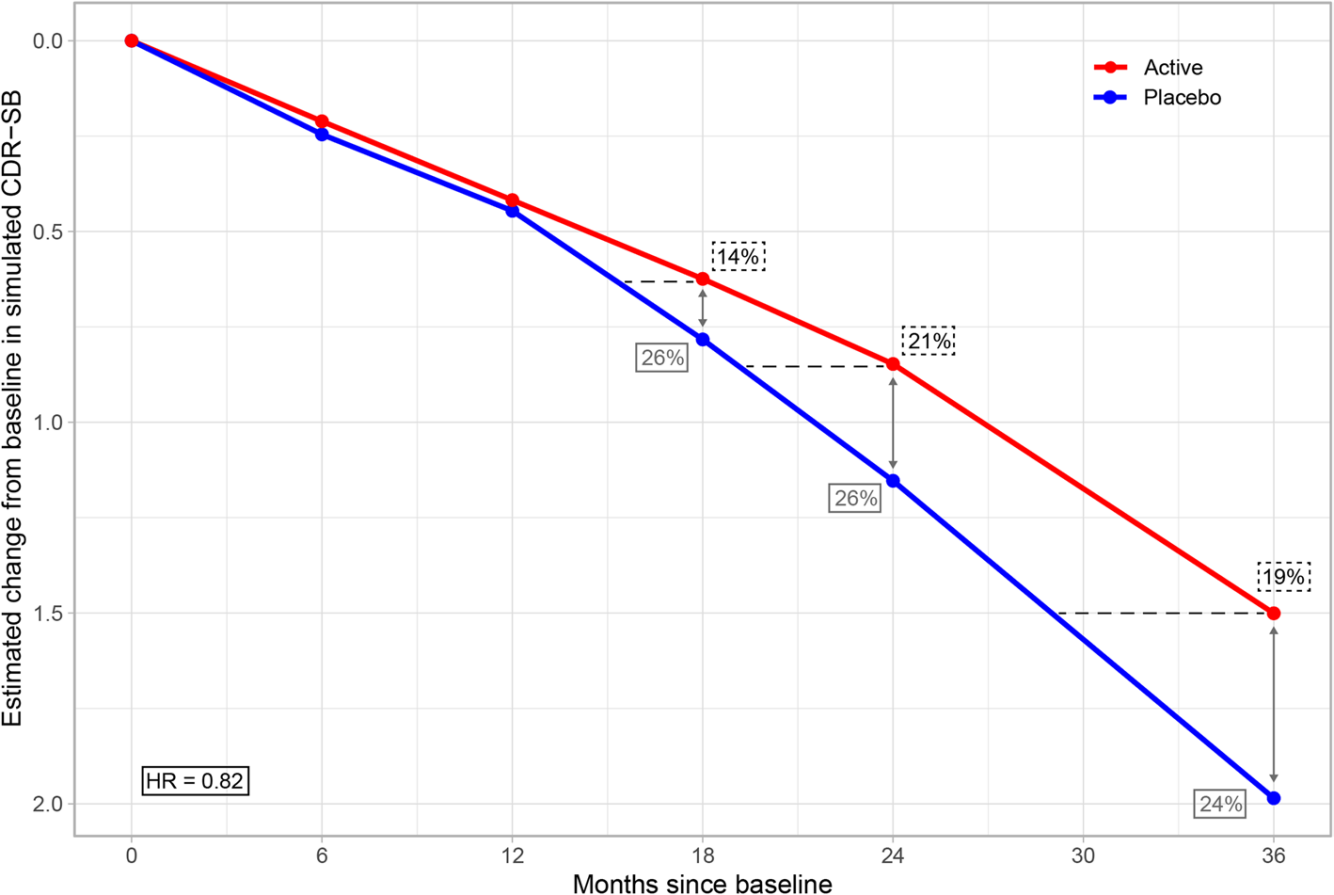
Estimated mean trajectory of the change from baseline in CDR-SB in a representative simulated scenario. Trajectories are shown for the PMRM and the MMRM models in a single simulated scenario with 700 subjects and a 36-month trial duration. The estimated trajectories for the PMRM and the MMRM were visually indistinguishable and are shown as a single trajectory within each treatment group (results may therefore deviate slightly from the medians shown in Table 2). The estimated hazard ratio from the Cox proportional model is shown in the bottom-left corner. The delay in disease progression, as estimated by the PMRM, is illustrated by the horizontal dotted lines, and the difference in CDR-SB, as estimated by the MMRM, is represented by the vertical arrows

Estimated mean trajectories of the change from baseline in CDR-SB from a single simulation of a scenario with 700 subjects and 36 months of follow-up are shown in Fig. 2 for illustrative purposes. The probability of progressing to dementia estimated by the Cox proportional hazards model (in this single scenario) is visualized in the Additional file.

All three models showed relatively consistent results across scenarios when estimating the treatment effect at the final visit with varying sample sizes and study lengths. The estimates from the PMRM ranged between 19 to 20% time delay in disease progression, the MMRM ranged between 22 to 26% in the difference in CDR-SB, and the Cox model estimated a hazard ratio of progressing to dementia between 0.82 and 0.85 (Table 2).

### Comparison of the statistical power

The PMRM was shown to consistently have a slightly greater power to detect treatment effects than that of MMRM, regardless of sample size and trial length. The Cox proportional hazards models would require a greater sample size to consistently detect treatment effects, only exceeding a power above 60% in the simulation with 700 patients and a trial length of 36 months. The results are presented in the Additional file.

### Implementation in health economic model

In the health economic model, the slowing of progression (PMRM) effect was implemented by extending the time axis of the active arm by 19% (based on the estimated median time delay from the 18 months scenarios in Table 2) and assuming transitions occur at these revised time points (1.19, 2.38, 3.57, 4.76… months, etc.). The slowing was assumed to persist throughout the time horizon, at 19%, and therefore indirectly continue to slow progression from all states including to death. This resulted in a delay of transitions at each month when compared to the placebo arm.

**Fig. 3:**
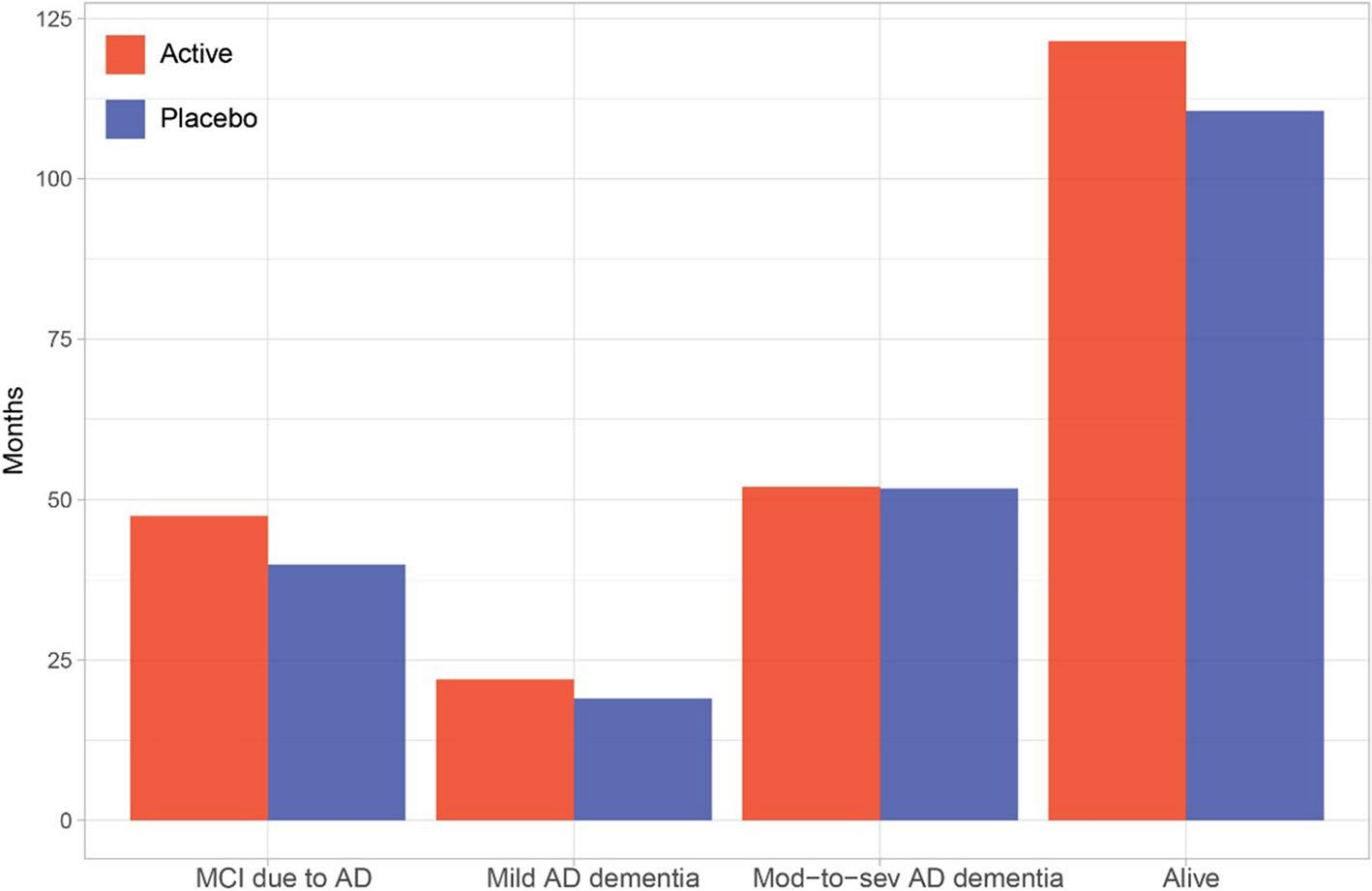
Average time spent in each health state including overall life expectancy over 15 years. The figure includes a comparison of placebo and treatment arm simulations

The modeling suggested an average overall survival of 111 and 121 months for the placebo and treatment arms, respectively. This implies estimated life expectancy gains of 10 months for treated patients compared to placebo. Treated patients were estimated to spend an additional 7 months in MCI due to AD and, additionally, 3 months in mild AD dementia, compared to placebo, whereas both arms spent an equal amount of time in moderate to severe AD dementia (Fig. 3).

## Discussion

In this modeling simulation study, we explored how estimates on the slowing of disease progression can be derived from clinical trial data and be used in a health-economic model for exploring long-term outcomes of disease modification in AD.

First, we showed that PMRM methods can accurately represent a hypothetical slowing of progression at 20%, translating this to a 7-month delay of progression over a trial duration of 36 months. According to the MMRM model, such slowing in our simulated scenarios corresponded to a reduction in the decline of CDR-SB over 36 months of about 25% or approximately 0.5 points on the CDR-SB. Arguably, reporting a treatment effect in terms of the number of months gained at a certain point in time on a global function measure such as CDR-SB represents a face-valid and readily interpretable approach for clinicians, patients, and families alike, compared to points gained on such measure [23]. The PMRM methods therefore enable translation of clinical trial endpoints to a metric (time) that can be perceived in clinically meaningful terms by patients, their caregivers, and health care professionals in general [53]. Reporting on the slowing of disease may be better aligned with the underlying mode of action of a DMT where much of the benefit is expected to accrue in the long term, in contrast to symptomatic treatments which need to demonstrate acute benefits in the short term. Nevertheless, this requires assumptions on the persistence of effects over the long term. Furthermore, estimating a time component using PMRM makes it possible to assess different aspects of disease on a common time scale, facilitating comparisons across outcomes (both within and between trials) [54]. This could be part of a multidimensional reporting (i.e., with a broader use of multiple outcomes and their representations) of clinical trial data and help contextualize the potential benefits of treatments as suggested by others [28, 55].

Second, we showed that PMRM methods had slightly greater power across all scenarios compared to MMRM for estimating a treatment effect which is a slowing of disease progression. This power gain is likely the result of the treatment effect at the final visit being computed using data from multiple visits in the placebo arm due to the spline interpolation. Another potential factor may be that the space of possible trajectories of PMRM is slightly lower than that of MMRM (i.e., the active arm cannot improve beyond the range of placebo trajectories without awkward extrapolation). This may contribute to the slight advantage for PMRM which we are seeing. The low power of the Cox models is probably due to the fact that they rely on a relatively simple dichotomous outcome, thereby disregarding the granularity offered by CDR-SB and disregarding multiple observations over time (as they are summarized in a single time to event). Furthermore, for the Cox models, no effort was made to account for the interval-censored data that arise in trials with visits occurring on a small number of discrete time points. The implications of using a model with a higher power to detect treatment effects may be that fewer subjects are needed in clinical trial, which would improve its feasibility and lower its cost.

Third, we showed that estimates of slowing of progression can be implemented in a standard Markov model which are commonly used for the health economic evaluation of healthcare interventions. By manipulating the time signatures on the *x*-axis, we allowed the treatment arm to progress at a slower rate (19% as estimated with the PMRM) and compared the results to the natural progression expected for patients on placebo. We chose the estimated effect (rather than the true 20% effect) to reflect the fact that the true effect is typically not known. Given the assumptions of our simple health economic model, a delay of progression of 7 months at the end of the 36-month trial is expected to result in an additional 10 months of life expectancy, 7 months without progressing from MCI due to AD to more severe states and 3 additional months in mild AD dementia. These estimates rely on the assumption that the slowing of 19% would persist over time, irrespective of in which states patients are currently in, and it ultimately results in an equal delay in time to death. The approach can also be extended to more complex models, e.g., with time-varying transition intensities, and with more flexible implementation of the treatment effect, e.g., to allow for different assumptions regarding treatment effects on mortality.

The results of the present study are in concordance with the study by Raket (2022) which compared the PMRM model with conventional models to quantify treatment effects in terms of slowing of disease progression using both simulated and historical data from AD clinical trials. However, there were a few differences, the first being the choice of primary outcome (cognitive subscale of the AD assessment scale) and the second being the competing model (constrained longitudinal data analysis) [36]. Two recently published studies also emphasized the importance of time-based approaches for assessing the effect of a DMT, donanemab [54, 56]. The study by Dickson et al., 2023 used data from the TRAILBLAZER-ALZ study and reported a delay in progression of disease by 5.3 months and 5.2 months as measured by the Integrated Alzheimer’s Disease Rating Scale (iADRS) and the CDR-SB, respectively after 18 months of treatment. In addition, the study analyzed the TRAILBLAZER-ALZ dataset using time-based PMRM methodology, resulting in similar findings (delay by 5.4–5.8 months) [54]. The other study by Sims et al., 2023 was the TRAILBLAZER-ALZ 2 study which also reported a delay in disease progression by 4.36 (95% CI, 1.87–6.85) months and 7.53 (95% CI, 5.69–9.36) months on the iADRS and the CDR-SB, respectively, at 76 weeks in the low/medium tau population [56].

ADNI data may not be representative to other groups of patients with AD. For instance, differences in clinical definitions, inclusion criteria and geographical distribution of study sites may influence the average rate of cognitive decline observed in a study [57, 58]. While this may affect our estimated rates of decline, it should not have any important impact on our main findings.

## Limitations

Our analysis is limited by only simulating one type of treatment effect, i.e., a proportional 20% slowing of progression at each visit [36], however, evaluated PMRM across five different types of simulated treatment effects (including reduction in decline and stable benefit scenarios) and found the PMRM methods had an advantage in terms of power, when the treatment effects increased in time.

We used a simplified Markov model with constant transition intensities to demonstrate how estimates on slowing of progression can be implemented. As such we made several assumptions which may have impacted our results. For instance, we assumed treatment would have an impact on all causes of mortality which is likely not accurate [2], and we assumed the treatment effect persisted over time which would need to be confirmed in long-term studies.

## Conclusions

In conclusion, our study adds to the knowledge base on multidimensional reporting from clinical trials in AD. It shows how PMRM methods can be used to estimate treatment effects in terms of slowing of progression which translate to time metrics that can be readily interpreted and appreciated as meaningful outcomes for patients, care partners, and health care practitioners. Our modeling approaches also subsequently inform on how these findings can be incorporated into health economic analysis. Future studies may explore PMRM applications with actual clinical trial data and in other diseases.

### Supplementary Information

**Additional file 1.** Simulation of treatment effects. Single simulation – Cox proportional hazards model. Comparison of statistical power.

## Data Availability

No datasets were generated or analysed during the current study.

## Abbreviations

AD: Alzheimer’s disease
ADNI: Alzheimer’s Disease Neuroimaging Initiative
AFT: Accelerated failure time
CDR: Clinical dementia rating
CDR-SB: Clinical dementia rating scale–sum of boxes
CMS: Centers for Medicare & Medicaid Services
DMT: Disease-modifying treatment
FDA: Food and Drug Administration
iADRS: Integrated Alzheimer’s Disease Rating Scale
MCI: Mild cognitive impairment
MMRM: Mixed model for repeated measures
MMSE: Mini-Mental State Examination
PMRM: Progression model for repeated measures
QoL: Quality of life
US: United States

## References

[1] Wattmo C, Londos E, Minthon L (2014). Risk factors that affect life expectancy in Alzheimer’s disease: a 15-year follow-up. Dement Geriatr Cogn Disord.

[2] Aye S, Jonsson L, Gustavsson A, Tate A, Ptacek SG, Eriksdotter M (2023). Effect of mortality in cost-effectiveness modeling of disease-modifying treatment for Alzheimer’s disease. Alzheimers Dement (Amst).

[3] Collaborators GBDD (2019). Global, regional, and national burden of Alzheimer’s disease and other dementias, 1990–2016: a systematic analysis for the Global Burden of Disease Study 2016. Lancet Neurol.

[4] Gustavsson A, Raket LL, Lilja M, Rutten-Jacobs L, Fues Wahl H, Bagijn M (2021). Health utility in preclinical and prodromal Alzheimer’s disease for establishing the value of new disease-modifying treatments-EQ-5D data from the Swedish BioFINDER study. Alzheimers Dement.

[5] Brodaty H, Donkin M (2009). Family caregivers of people with dementia. Dialogues Clin Neurosci.

[6] Demirbas M, Hahn-Pedersen JH, Jørgensen HL (2023). Comparison between burden of care partners of individuals with Alzheimer’s disease versus individuals with other chronic diseases. Neurol Ther.

[7] Frederiksen KS, Lanctôt KL, Weidner W, Hahn-Pedersen JH, Mattke S. A Literature Review on the Burden of Alzheimer's Disease on Care Partners. J Alzheimers Dis. 2023;96(3):947–66. 10.3233/JAD-230487. 10.3233/JAD-230487 37980660

[8] Wimo A, Seeher K, Cataldi R, Cyhlarova E, Dielemann JL, Frisell O (2023). The worldwide costs of dementia in 2019. Alzheimers Dement.

[9] El-Hayek YH, Wiley RE, Khoury CP, Daya RP, Ballard C, Evans AR (2019). Tip of the Iceberg: assessing the global socioeconomic costs of Alzheimer’s disease and related dementias and strategic implications for stakeholders. J Alzheimers Dis.

[10] Gustavsson A, Norton N, Fast T, Frolich L, Georges J, Holzapfel D (2023). Global estimates on the number of persons across the Alzheimer’s disease continuum. Alzheimers Dement.

[11] Cummings J, Lee G, Zhong K, Fonseca J, Taghva K (2021). Alzheimer’s disease drug development pipeline: 2021. Alzheimers Dement (N Y).

[12] Cummings J, Zhou Y, Lee G, Zhong K, Fonseca J, Cheng F (2023). Alzheimer’s disease drug development pipeline: 2023. Alzheimers Dement (N Y).

[13] Atri A (2019). Current and future treatments in Alzheimer’s disease. Semin Neurol.

[14] Ngandu T, Lehtisalo J, Solomon A, Levalahti E, Ahtiluoto S, Antikainen R (2015). A 2 year multidomain intervention of diet, exercise, cognitive training, and vascular risk monitoring versus control to prevent cognitive decline in at-risk elderly people (FINGER): a randomised controlled trial. Lancet.

[15] Food and Drug Administration. FDA grants accelerated approval for Alzheimer’s drug. FDA News Release. 2021. https://www.fda.gov/news-events/press-announcements/fda-grants-accelerated-approval-alzheimers-drug. Accessed 25 Apr 2023.

[16] Food and Drug Administration. FDA converts novel Alzheimer’s disease treatment to traditional approval: action follows confirmatory trial to verify clinical benefit. 2023. https://www.fda.gov/news-events/press-announcements/fda-converts-novel-alzheimers-disease-treatment-traditional-approval. Accessed 7 Sept 2023.

[17] Centers for Medicare and Medicaid Services. https://www.cms.gov/newsroom/press-releases/cms-finalizes-medicare-coverage-policy-monoclonal-antibodies-directed-against-amyloid-treatment. Accessed 7 Sept 2023.

[18] Brockmann R, Nixon J, Love BL, Yunusa I (2023). Impacts of FDA approval and Medicare restriction on antiamyloid therapies for Alzheimer’s disease: patient outcomes, healthcare costs, and drug development. Lancet Reg Health Am.

[19] Centers for Medicare & Medicaid Services C. Statement: broader medicare coverage of leqembi available following FDA traditional approval. https://www.cms.gov/newsroom/press-releases/statement-broader-medicare-coverage-leqembi-available-following-fda-traditional-approval. Accessed 7 Sept 2023.

[20] Mattke S, Hanson M. Expected wait times for access to a disease-modifying Alzheimer's treatment in the United States. Alzheimers Dement. 2022;18(5):1071–4. 10.1002/alz.12470. 10.1002/alz.12470 34569686

[21] Cummings J, Aisen P, Lemere C, Atri A, Sabbagh M, Salloway S (2021). Aducanumab produced a clinically meaningful benefit in association with amyloid lowering. Alzheimers Res Ther.

[22] Wolk DA, Rabinovici GD, Dickerson BC (2023). A step forward in the fight against dementia-are we there yet?. JAMA Neurol.

[23] Petersen RC, Aisen PS, Andrews JS, Atri A, Matthews BR, Rentz DM (2023). Expectations and clinical meaningfulness of randomized controlled trials. Alzheimers Dement.

[24] Reiman EM (2023). Drug trial for Alzheimer’s disease is a game changer. Nature.

[25] Mullard A (2021). Landmark Alzheimer’s drug approval confounds research community. Nature.

[26] Rubin R (2021). Recently approved Alzheimer drug raises questions that might never be answered. JAMA.

[27] Walsh S, Merrick R, Milne R, Brayne C (2021). Aducanumab for Alzheimer’s disease?. BMJ.

[28] Assuncao SS, Sperling RA, Ritchie C, Kerwin DR, Aisen PS, Lansdall C (2022). Meaningful benefits: a framework to assess disease-modifying therapies in preclinical and early Alzheimer’s disease. Alzheimers Res Ther.

[29] Gustavsson A, Green C, Jones RW, Forstl H, Simsek D, de Reydet de Vulpillieres F (2017). Current issues and future research priorities for health economic modelling across the full continuum of Alzheimer’s disease. Alzheimers Dement.

[30] Ellison TS, Cappa SF, Garrett D, Georges J, Iwatsubo T, Kramer JH (2023). Outcome measures for Alzheimer’s disease: a global inter-societal Delphi consensus. Alzheimers Dement.

[31] Kahle-Wrobleski K, Ye W, Henley D, Hake AM, Siemers E, Chen YF (2017). Assessing quality of life in Alzheimer’s disease: implications for clinical trials. Alzheimers Dement (Amst).

[32] Andrews JS, Desai U, Kirson NY, Zichlin ML, Ball DE, Matthews BR (2019). Disease severity and minimal clinically important differences in clinical outcome assessments for Alzheimer’s disease clinical trials. Alzheimers Dement (N Y).

[33] Cohen S, Cummings J, Knox S, Potashman M, Harrison J (2022). Clinical trial endpoints and their clinical meaningfulness in early stages of Alzheimer’s disease. J Prev Alzheimers Dis.

[34] Petersen RC, Thomas RG, Grundman M, Bennett D, Doody R, Ferris S (2005). Vitamin E and donepezil for the treatment of mild cognitive impairment. N Engl J Med.

[35] Rentz DM, Wessels AM, Annapragada AV, Berger AK, Edgar CJ, Gold M (2021). Building clinically relevant outcomes across the Alzheimer’s disease spectrum. Alzheimers Dement (N Y).

[36] Raket LL (2022). Progression models for repeated measures: Estimating novel treatment effects in progressive diseases. Stat Med.

[37] Briggs A, Sculpher M (1998). An introduction to Markov modelling for economic evaluation. Pharmacoeconomics.

[38] Handels RLH, Green C, Gustavsson A, Herring WL, Winblad B, Wimo A (2023). Cost-effectiveness models for Alzheimer’s disease and related dementias: IPECAD modeling workshop cross-comparison challenge. Alzheimers Dement.

[39] Karimi A, Delpisheh A, Sayehmiri K (2016). Application of accelerated failure time models for breast cancer patients’ survival in Kurdistan Province of Iran. J Cancer Res Ther.

[40] Swindell WR (2009). Accelerated failure time models provide a useful statistical framework for aging research. Exp Gerontol.

[41] Alzheimer’s Disease Neuroimaging initiative. https://adni.loni.usc.edu/. Accessed 17 Aug 2023.

[42] Raket LL (2020). Statistical disease progression modeling in Alzheimer disease. Front Big Data.

[43] Folstein MF, Robins LN, Helzer JE (1983). The mini-mental state examination. Arch Gen Psychiatry.

[44] McDougall F, Edgar C, Mertes M, Delmar P, Fontoura P, Abi-Saab D (2021). Psychometric properties of the clinical dementia rating - sum of boxes and other cognitive and functional outcomes in a prodromal Alzheimer’s disease population. J Prev Alzheimers Dis.

[45] O’Bryant SE, Lacritz LH, Hall J, Waring SC, Chan W, Khodr ZG (2010). Validation of the new interpretive guidelines for the clinical dementia rating scale sum of boxes score in the national Alzheimer’s coordinating center database. Arch Neurol.

[46] Fitzmaurice GM, Laird NM, Ware JH. Applied longitudinal analysis. Wiley; 2012.

[47] Cox DR (1972). Regression models and life-tables. J Roy Stat Soc: Ser B (Methodol).

[48] Detry MA, Ma Y (2016). Analyzing repeated measurements using mixed models. JAMA.

[49] Wei L-J (1992). The accelerated failure time model: a useful alternative to the Cox regression model in survival analysis. Stat Med.

[50] Lin G, Whittington MD, Wright A, Agboola F, Herron-Smith S, Pearson SD, et al. Lecanemab for early Alzheimer’s disease. https://icer.org/wp-content/uploads/2023/04/ICER_Alzheimers-Disease_Final-Report_For-Publication_04172023.pdf. Accessed 7 Sept 2023. 10.18553/jmcp.2023.29.9.1078 PMC10512855 37610113

[51] Potashman M, Buessing M, Levitchi Benea M, Cummings J, Borson S, Pemberton-Ross P (2021). Estimating progression rates across the spectrum of Alzheimer’s disease for amyloid-positive individuals using National Alzheimer’s coordinating center data. Neurol Ther.

[52] Chhatwal J, Jayasuriya S, Elbasha E (2014). Changing cycle lengths in state-transition models: doing it the right way. ISPOR Connect.

[53] Tochel C, Smith M, Baldwin H, Gustavsson A, Ly A, Bexelius C (2019). What outcomes are important to patients with mild cognitive impairment or Alzheimer’s disease, their caregivers, and health-care professionals? A systematic review. Alzheimers Dement (Amst).

[54] Dickson SP, Wessels AM, Dowsett SA, et al. ‘Time Saved’ As a Demonstration of Clinical Meaningfulness and Illustrated Using the Donanemab TRAILBLAZER-ALZ Study Findings. J Prev Alzheimers Dis. 2023;10:595–9. 10.14283/jpad.2023.50 37357301

[55] Jessen F, Georges J, Wortmann M, Benham-Hermetz S (2022). What matters to patients with Alzheimer’s disease and their care partners? Implications for understanding the value of future interventions. J Prev Alzheimers Dis.

[56] Sims JR, Zimmer JA, Evans CD, Lu M, Ardayfio P, Sparks J (2023). Donanemab in early symptomatic Alzheimer disease: the TRAILBLAZER-ALZ 2 randomized clinical trial. JAMA.

[57] Berres M, Monsch AU, Spiegel R (2021). Using historical data to facilitate clinical prevention trials in Alzheimer disease? An analysis of longitudinal MCI (mild cognitive impairment) data sets. Alzheimer’s Res Ther.

[58] Cummings JL, Atri A, Ballard C, Boneva N, Frölich L, Molinuevo JL (2018). Insights into globalization: comparison of patient characteristics and disease progression among geographic regions in a multinational Alzheimer’s disease clinical program. Alzheimers Res Ther.

