# Supplementary material for: Progression analysis versus traditional methods to quantify slowing of disease progression in Alzheimer’s disease
Source: Alzheimers Res Ther. 2024 Feb 29;16:48. doi: 10.1186/s13195-024-01413-y (PMC10903002; doi:10.1186/s13195-024-01413-y)
Supplement: Supplementary file 1 — Additional file 1. Simulation of treatment effects. Single simulation – Cox proportional hazards model. Comparison of statistical power. [file 13195_2024_1413_MOESM1_ESM.docx]

**Additional file:**

**Simulation of treatment effects**

Data in the simulation study were simulated from the following model:

$$y_{ij}=\alpha_{j,\text{treatment}\left( i \right)}+e_{ij}$$

where

$$\boldsymbol{\alpha}_{\text{placebo}}=\left( \alpha_{j,\text{placebo}} \right)_{j=0,\ldots,5}=\left( 1.52, 1.79, 2.01, 2.31, 2.69, 3.53 \right)$$

and

$${(e_{ij})}_{j=0,\ldots,5}\sim N_{6}\left( 0,\boldsymbol{R} \right)$$

with

$$\boldsymbol{R=}\left( \begin{matrix} 0.79 & 0.74 & 0.82 & 0.97 & 1.10 & 1.39 \\ 0.74 & 1.44 & 1.36 & 1.60 & 1.75 & 2.36 \\ 0.82 & 1.36 & 2.05 & 2.10 & 2.48 & 3.39 \\ 0.97 & 1.60 & 2.10 & 2.93 & 3.23 & 4.52 \\ 1.10 & 1.75 & 2.48 & 3.23 & 4.89 & 6.10 \\ 1.39 & 2.36 & 3.39 & 4.52 & 6.10 & 10.41 \end{matrix} \right).$$

Let $\vartheta\left( t \right)$ denote the linear interpolation at time point $t$ of the mean values $\boldsymbol{\alpha}_{\text{placebo}}$ at the associated visit times ($t_{j}=$0, 6, 12, 18, 24 and 36 months since baseline). Treatment effects in the simulated active arm corresponding to a 20% delay in progression were generated as follows.

$$\left( \alpha_{j,\text{active}} \right)_{j=0,\ldots,5}=\left( \vartheta\left( 0.8\cdot t_{j} \right) \right)_{j=0,\ldots,5}.$$

The mean value of the placebo arm and the active arm are given in the table below.

|  | **Visit (month)** | | | | | |
| --- | --- | --- | --- | --- | --- | --- |
| **CDR-SB** | **Baseline** | **6** | **12** | **18** | **24** | **36** |
| Placebo | 1.52 | 1.79 | 2.01 | 2.31 | 2.69 | 3.53 |
| Active treatment | 1.52 | 1.73 | 1.92 | 2.13 | 2.39 | 3.03 |

**Single simulation – Cox proportional hazards model**

The figure shows the probability of progression to dementia and hazard ratio (HR) estimated by the Cox proportional hazards model in a single simulation of a scenario with 700 subjects and 36 month trial duration.

**
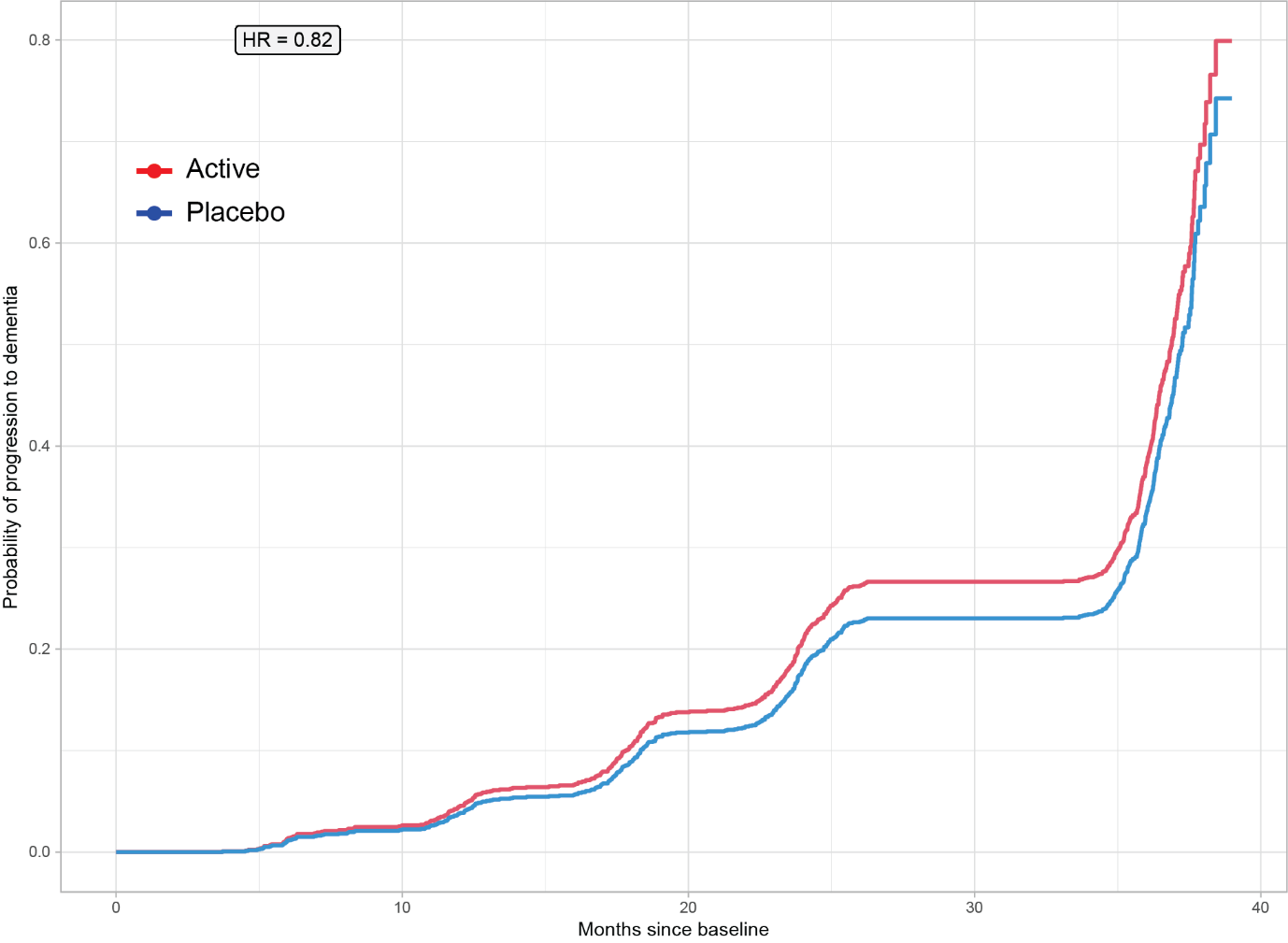
**

**Comparison of statistical power**

The statistical power of PMRM, MMRM and Cox proportional hazard model across trial scenarios has been compared in the figure and the table given below:

**
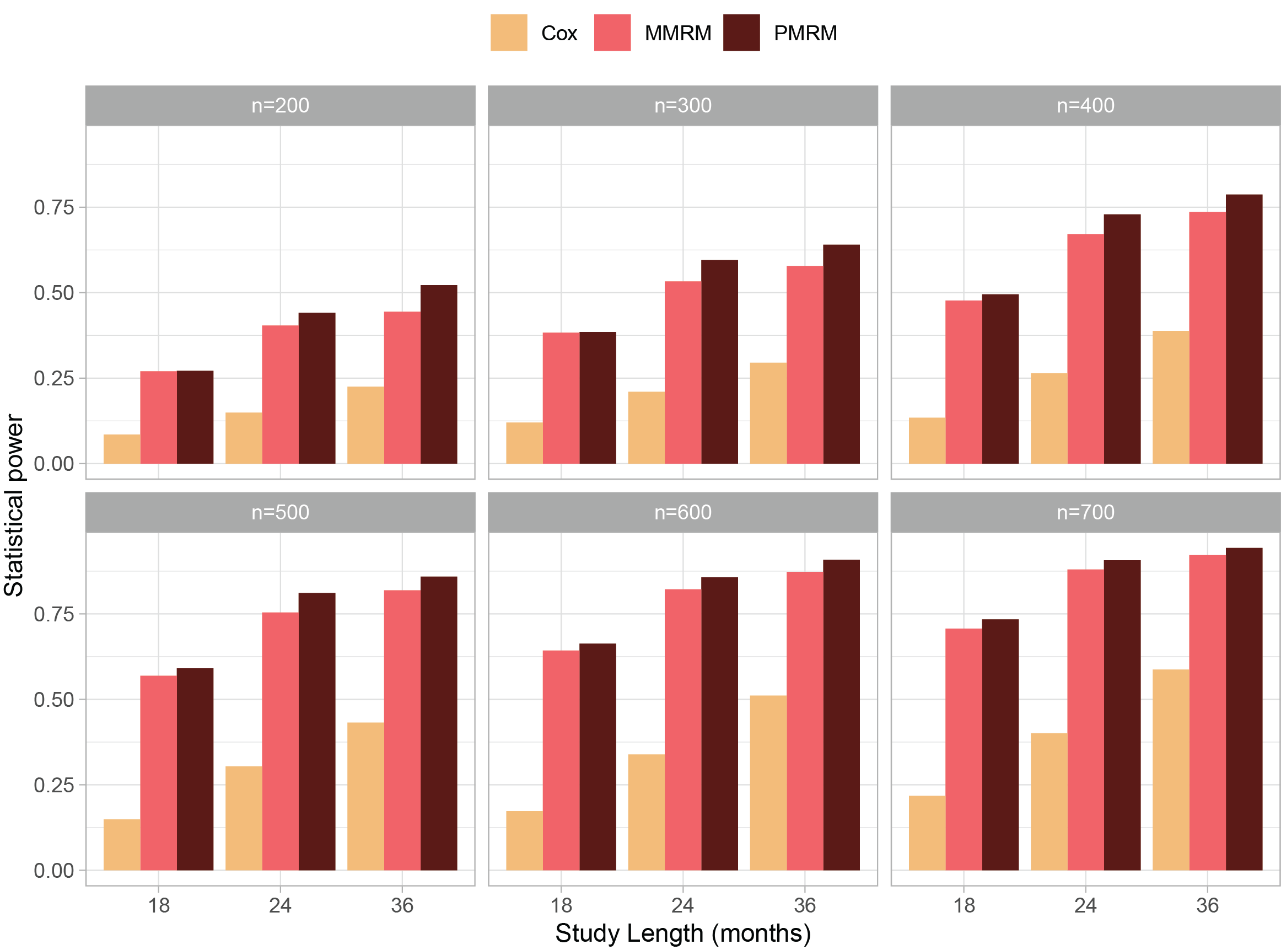
**

| **Patients per arm** | **Study length (months)** | **PMRM** | **MMRM** | **Cox** |
| --- | --- | --- | --- | --- |
| 200 | 18 | 0.271 | 0.270 | 0.085 |
| 200 | 24 | 0.441 | 0.404 | 0.149 |
| 200 | 36 | 0.522 | 0.444 | 0.225 |
| 300 | 18 | 0.385 | 0.383 | 0.120 |
| 300 | 24 | 0.595 | 0.533 | 0.210 |
| 300 | 36 | 0.640 | 0.578 | 0.295 |
| 400 | 18 | 0.495 | 0.477 | 0.134 |
| 400 | 24 | 0.729 | 0.671 | 0.264 |
| 400 | 36 | 0.787 | 0.736 | 0.388 |
| 500 | 18 | 0.591 | 0.569 | 0.149 |
| 500 | 24 | 0.811 | 0.754 | 0.304 |
| 500 | 36 | 0.859 | 0.819 | 0.432 |
| 600 | 18 | 0.663 | 0.643 | 0.173 |
| 600 | 24 | 0.857 | 0.822 | 0.339 |
| 600 | 36 | 0.908 | 0.873 | 0.511 |
| 700 | 18 | 0.734 | 0.707 | 0.218 |
| 700 | 24 | 0.907 | 0.880 | 0.401 |
| 700 | 36 | 0.943 | 0.922 | 0.587 |
